# Facile Synthesis and Electrochemical Studies of Mn_2_O_3_/Graphene Composite as an Electrode Material for Supercapacitor Application

**DOI:** 10.3389/fchem.2021.717074

**Published:** 2021-08-18

**Authors:** Ghulam Mustafa, Gohar Mehboob, Said Nasir Khisro, Muhammad Javed, Xinman Chen, M. Shafiq Ahmed, J. M. Ashfaq, G. Asghar, Shahnwaz Hussain, Amin ur Rashid, Ghazanfar Mehboob

**Affiliations:** ^1^ Department of Physics, University of Kotli, Kotli, Pakistan; ^2^ School of Materials Science and Engineering, South China University of Technology, Guangzhou, China; ^3^ Institute of Optoelectric Materials and Technology, South China Normal University, Guangzhou, China; ^4^ Department of Physics, University of Poonch Rawlakot, Rawalakot, Pakistan; ^5^ State Key Laboratory for Mechanical Behavior of Materials, School of Materials Science and Engineering, Xi’an Jiatong University, Xi’an, China; ^6^ Department of Applied Physical and Material Sciences, University of Swat, Swat, Pakistan

**Keywords:** sol-gel method, graphene, Mn_2_O_3_, nickel foam, cyclic voltammetry

## Abstract

A simplified sol-gel method that can be scaled up for large-scale production was adopted for the preparation of manganese oxide nanocrystals. Prepared Mn_2_O_3_ exhibited micron-sized particles with a nanoporous structure. In the present study, a simple and low-cost strategy has been employed to fabricate nanoporous Mn_2_O_3_ with an increased surface area for an electrode/electrolyte interface that improved the conduction of Mn_2_O_3_ material. The crystal phase and morphology of the prepared material was investigated by X-ray diffraction (XRD), scanning electron microscopy (SEM), and energy-dispersive X-ray spectroscopy (EDX). The prepared electrode materials were deposited on a nickel foam substrate to investigate the electrochemical properties. The galvanostatic charge/discharge (GCD), cyclic voltammetry (CV), and complex impedance studies confirmed excellent specific capacitance and capacitive behavior of the prepared material. The synthesized Mn_2_O_3_/graphene composites exhibited an excellent specific capacitance of 391 F/g at a scan rate of 5 mV/S. Moreover, a specific capacitance of 369 F/g was recorded at a current density of 0.5 A/g using the galvanostatic charge/discharge test. The high porosity of the materials provided a better electrolyte-electrode interface with a larger specific area, thus suggesting its suitability for energy storage applications.

## Introduction

Over the past few decades, energy storage strategies are one of the key challenges owing to the progressive exhaustion of fossil fuels and a fast energy depletion rate (Serrapede et al., 2019). Renewable energy generation from different natural resource such as the wind, tides, and sun is noncontrollable, noncontinuous, and volatile because it largely depends on environmental conditions. As a consequence, the energy storage technologies need to be enhanced with an improved efficiency that might be more environmental friendly (Rehman et al., 2016; 2017). Energy storage systems comprise of capacitors, fuel cells, electrochemical supercapacitors, and batteries. Among these systems, supercapacitors deliver high efficiency, high power density, improved cycle life (>100,000 cycles), a large range of working temperature, and extended self-life (Rafique et al., 2017a).

To make an active electrode material for supercapacitors various materials have been exploited, for instance, activated carbon (Chen et al., 2016a), carbon nanotubes (Hulicova Jurcakova et al., 2009), carbon quantum dots (Chen et al., 2016b; Shi et al., 2020), carbon onions (Portet et al., 2007), carbon fibers (Wu et al., 2010; Luo et al., 2012; Chen et al., 2016a) and other carbon-based materials (Lamberti et al., 2016a; 2017), electric polymer conductors (Wang et al., 2012; Shi et al., 2015), and transition metal ceramic oxides (Nagarajan and Zhitomirsky, 2006; Aghazadeh, 2012; Rafique et al., 2017a). However, the main problem that limits the usage of carbonaceous materials in supercapacitors arises due to low specific capacitance, low energy density storage per cycle (Lamberti et al., 2016b), and electrode stability and regarded as costly when noble metal oxides are employed (Cao et al., 2015). Recently, studies have been carried out for the development of low-cost alternative materials (Nithya et al., 2015) such as manganese oxide and iron oxide as a substitute for ruthenium oxides (Zhi et al., 2013). In previous studies, high-cost and complex methods were used to get the desired capacitance for graphene oxide/Mn_2_O_3_ composite electrode on Ni foam, while in the present study effective and simple techniques are used to get the valuable electrochemical results (Han et al., 2020). The high specific capacitance and environment friendly behavior of Mn_2_O_3_ attracted much attention of the researcher to use it for supercapacitors. However, a low surface area and poor conductivity hinder it to use as an electrode material (Lu et al., 2020).

In the recent past, lot of efforts have been made to synthesize a composite material to exploit the properties of both carbonaceous (high conductivity) and transition metal oxides (TMOs) (high specific capacitance) such as a manganese oxide composite with carbon nanofoams (Fischer et al., 2007), carbon fibers, exfoliated graphite, and carbon nanotubes. Theoretical studies suggest that manganese oxide-based supercapacitors have high capacitance (approx. 1,400 Fg^-1^). Mn_2_O_3_ is an inexpansive, environmental friendly, naturally abundant material (Luo et al., 2012; Pirri et al., 2017). The composites of these two materials offer a synergic effect and improve electrochemical performance of the device (Wu et al., 2010). Herein, the present study aims to prepare electrode material of Mn_2_O_3_/graphene composites *via* a simple physical deposition method and characterize the structural properties of Mn_2_O_3_/graphene composites powder by X-ray diffraction (XRD) and the microstructural features by scanning electron microscopy (SEM). The elemental study was performed by an energy-dispersive X-ray spectroscopy (EDX). The main focus of the present study was to investigate the electrochemical performance of prepared composite materials. In the present study, the electrochemical performance demonstrates that Mn_2_O_3_/graphene nanocompsites have great potential with a simple and low-cost method as compared to report previous.

## Experimental Procedures

### Synthesis of Mn_2_O_3_ Nanoparticles

The sol-gel method was used (Nasir et al., 2011) to synthesize Mn_2_O_3_ nanoparticles by dissolving 25 gm of manganese nitrate (Mn(NO_3_)_2_.4H_2_O) in 100 ml of ethylene glycol. The amount of the solvent was adjusted such that manganese nitrate was completely dissolved. The solution was further heated at 80°C under continuous stirring until a thick gel was formed. The gel burnt slowly into a fine powder by increasing temperature to 100°C. The resultant powder was sintered at 700°C in an electric furnace for 4 hours.

### Synthesis of Mn_2_O_3_/Graphene Composites

The composites of Mn_2_O_3_/graphene with the ratio (1:1, 1:2, and 1:3) were prepared by mixing appropriate amounts of the composition with a mortar and pestle for 30 min. Then the mixture was dispersed in deionized water and sonicated for 30 min hour. Afterward, the mixture was poured into an electric motor blender and was blended for 30 min. While blending, nanoporous Mn_2_O_3_ micro-particles having higher density than graphite acted as high-energy projectiles hitting graphite clusters and exfoliated them to graphene, thus improving uniformity and mixing of the composites. Afterward, a microwave oven has been employed to dry the obtained composite materials for 1 hour.

### Electrode Preparation

The Ni foam with an area of about (1 cm^2^ × 1 cm^2^) was used as a substrate. The deionized water was added to the mixture of composite material and PVA (10%) to make a thick slurry. This slurry of the composite material was then transferred into a glass tube and sonicated for 15 min. The substrate was inserted into the slurry and sonicated for 10 min such that the pores of the Ni foam (Han et al., 2020; Rabani et al., 2020) uniformly filled with the slurry. The substrate was then dried for 1 h in an oven. The mass of the electrode material was calculated by weighing Ni foam before and after loading the electrode material. Moreover, the electrochemical analysis of prepared electrodes was performed by using a CHI660E electrochemical workstation.

## Results and Discussion

The materials were explored for structural properties by employing the XRD technique. The crystal structure of the synthesized materials was investigated by employing an X-ray powder diffraction technique (XRD diffractometer, Panalytical X’Pert Pro) with Cu (K_α_) having a wavelength of 1.54 angstrom as an X-ray source.

**FIGURE 1:**
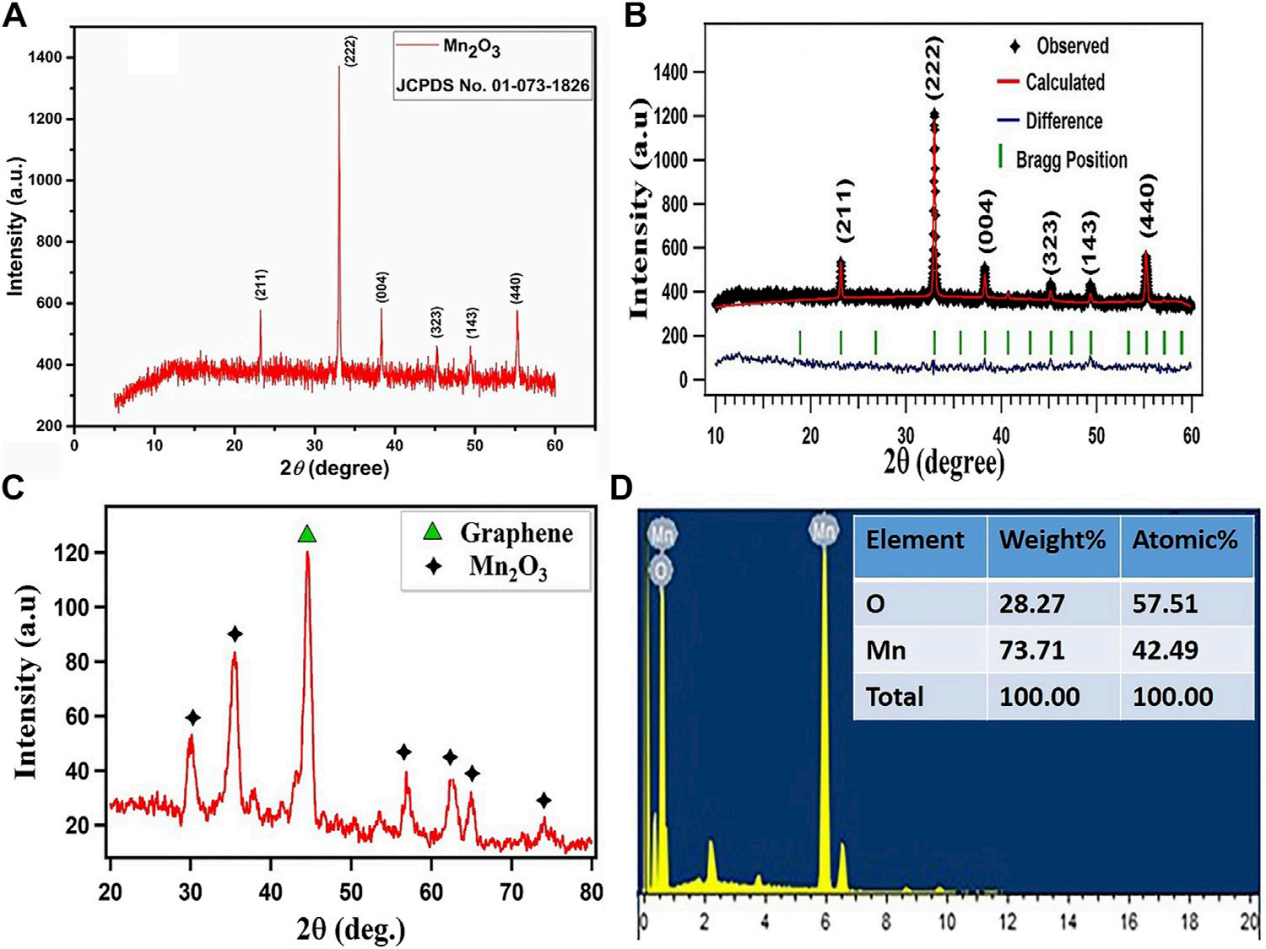
**(A,C)** XRD spectra of Mn_2_O_3_ nanoparticles and its composite with graphene **(B)** full proof refinement of Mn_2_O_3_ **(D)** EDX spectrum of Mn_2_O_3_ nanoparticles with compositional analysis in the inset.

The XRD data were obtained by scanning the samples for 2θ values ranging from 10° to 60°. Figure 1A depicts the XRD pattern profile of the single phase Mn_2_O_3_ ceramic. All the Bragg reflection peaks in the spectrum were matched well with the JCPDF card no. 01-073-1826 that correspond to the cubic structure of Mn_2_O_3_. The average crystallite size of Mn_2_O_3_ metal oxide ceramic was estimated from the most intense peak occurring at 2θ ∼ 32.92° that corresponds to (222) the plane of the cubic structure of Mn_2_O_3_ by using the Deby–Scherer equation, which was found to be 22.67 nm (Zhang et al., 2009; Lee, 2014). The XRD profile of Mn_2_O_3_ reveals well-defined and prominent Bragg reflection peaks which correspond to Mn_2_O_3_ stoichiometry of the compound (Tao et al., 2016; Rafique et al., 2017b).

The material was further investigated for the crystalline structure and a quantitative analysis, and the XRD profile of Mn_2_O_3_ was refined by Rietveld refinement executed by a FullProf Suite program, and is displayed in Figure 1B. The XRD profile is represented by the black symbols, the refined pattern by the red solid line, the positions of Bragg reflections by green vertical ticks, and the difference between the experimental and estimated patterns for Mn_2_O_3_ under investigation is illustrated by blue solid line. The pseudo-Voigt function defined in the background mode of a twelve coefficients Fourier cosine series has been used to model the powder profile refinement of the XRD pattern. Different parameters have been iterated such as preferred orientation (G), atom coordinates (*x, y, z*), cell geometrical parameters (α, β, γ) and (*a, b, c*), occupation (occ), thermal parameters (Bs), and FWHM parameters (U, V, W). The agreement between the experimental and approximated profiles was checked by the discrepancy factors which comprise the expected R-factor (R_exp_), the Bragg R-factor, the weighted profile R-factor (R_WP_), and goodness of fit (χ^2^). The XRD Rietveld refinement revealed a cubic unit cell with a space group symmetry of Ia-3 space and computed various structural parameters of the crystal structure for Mn_2_O_3_ particles. The summary of the refined results are enlisted in the table.

Figure 1C depicts the XRD spectrum of Mn_2_O_3_/graphene composite. Here, the black symbol (star) marks the Bragg reflections of the Mn_2_O_3_ single phase, while the high intensity diffraction peak appearing at 2θ value of 44.57° represented by green symbol (triangle) indicates the Bragg reflections arising from graphene. Thus, the XRD profile confirms the presence of both phases and assures the successful formation of Mn_2_O_3_/graphene composite.

The elemental composition of Mn_2_O_3_ has been examined by an energy-dispersive x-ray (EDX) technique, as shown in Figure 1D. The EDX results refer to the presence of all the expected elements in Mn_2_O_3_ and discard the presence of other elements; this assures the phase purity of the material and hence these results are consistent with the XRD data discussed earlier (Zhang et al., 2009; Gnanam and Rajendran, 2011; Wu et al., 2012).

**FIGURE 2:**
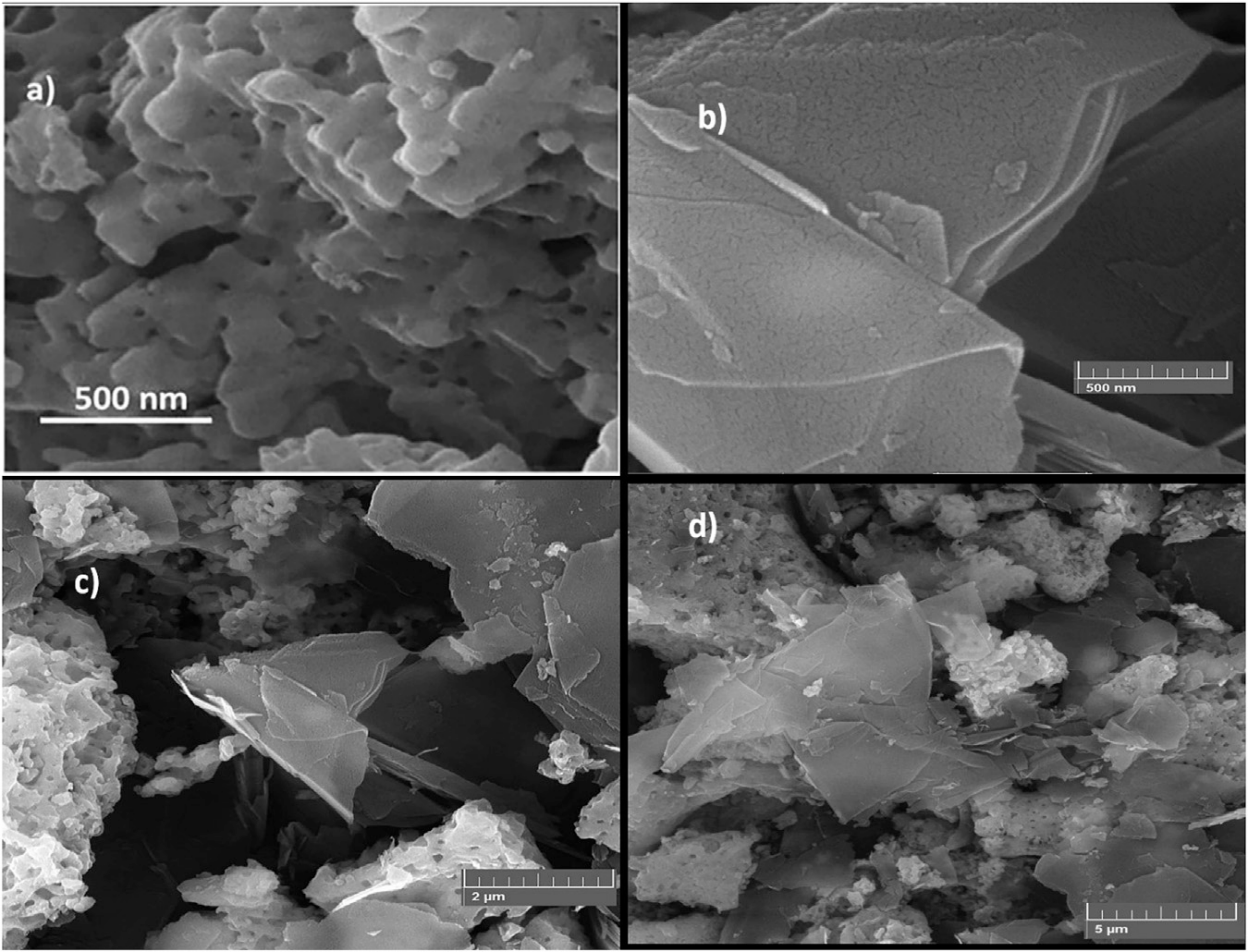
**(A–D)**: SEM image of **(A)** Mn_2_O_3_, **(B)** graphene, and **(C–D)** Mn_2_O_3_/graphene composite.

The morphology of both the single-phase Mn_2_O_3_ and Mn_2_O_3_/graphene composite was investigated by means of a scanning electron microscope (SEM) MIRA3 TESCAN, as shown in Figure 2. Figure 2A shows the SEM image of the sol-gel–derived Mn_2_O_3_ single phase. From the figure, it can be observed that surface morphology of Mn_2_O_3_ exhibits a nanoporous structure with agglomerated crystallites. The agglomeration may occur due to sintering of the material at higher temperature of about 700°C and also of magnetic nature of its nanoparticles (Bah et al., 2016). The surface morphology of graphene is displayed by a SEM image publicized in Figure 2B which reveals sheet-like microstructural texture with close stacking. Figures 2C,D displays the morphology of Mn_2_O_3_/graphene composite. Generally, the porosity of the composite is beneficial for ion transport to the electrode-electrolyte interface and improves the efficiency of the charge storage device (Shinde et al., 2013). The cracked area in the Figures 2C,D indicates the formation of a relatively dense surface layer; however, the inner layer has higher micro porosity. These microstructural features revealed smooth surfaces and discarded any amorphous coating. The crystallites exhibited nearly uniform diameters separated by sharply defined boundaries. The composite exhibits a sheet-like microstructural texture of graphene and with fluffy and porous Mn_2_O_3_ crystals embedded well between the exfoliated graphene sheets. It also indicates that the presence of Mn_2_O_3_ nanoparticle has no effect on the graphene sheets and composite exhibits almost uniform compositions (Sharrouf et al., 2015; Wang et al., 2020) which is very desirable for good electrochemical behavior of the material.

### Electrochemical Characterization

The galvanostatic charge discharge and cyclic moltamogram measurements in a three electrode setup have been used to study electrochemical performance of Mn_2_O_3_/graphene composite deposited on Nickel foam as an electrode material, where a saturated calomel electrode (SCE) as a reference electrode, platinum (Pt) is used as a counter electrode, and Mn_2_O_3_/graphene as a working electrode in 1 M K_2_SO_4_ as electrolyte.

**FIGURE 3:**
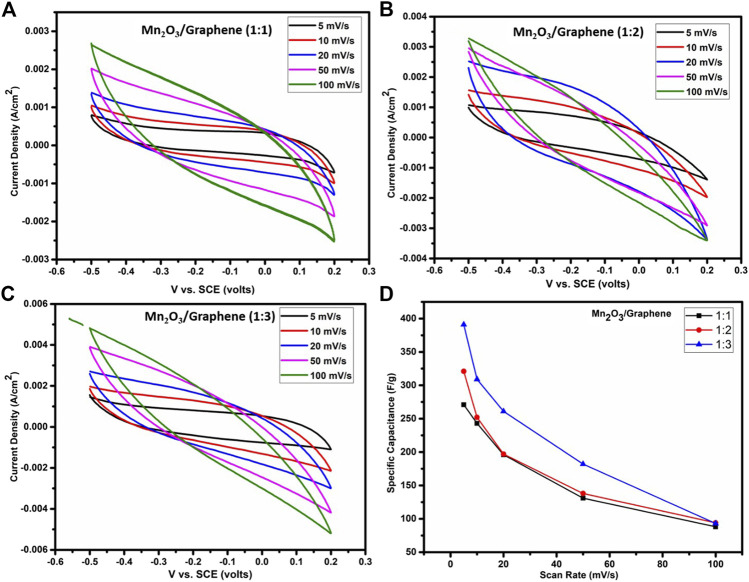
The CV’s recorded at a scan rate of 5 mV/s to 100 mV/s of Mn_2_O_3_/graphene composite for **(A)** (1:1), **(B)** 1:2, **(C)**1:3, and **(D)** comparison of specific capacitance of all three nanocomposites.

The cyclic voltammetric study performed at 5, 10, 20, 50, and 100 mV/s scan rates, respectively, for all three composites, as shown in Figures 3(A–C), which exhibits quasi-rectangular shape curves. These quasi-rectangular shapes of the composite material are closed to EDLC’s behavior, even though the faradaic processes are more dominating in the electrochemical behavior due to Mn_2_O_3_ nanoparticles in aqueous electrolyte indicative of pseudocapacitive behavior. The pseudo capacitance may arise from an Mn^+3^/Mn^+2^ couple. The below-mentioned equation was used to determine the specific capacitance of the cell (Rafique et al., 2017b):

$$C_{cell}=\frac{Q}{2V}=\frac{1}{2Vv}\overset{v+}{\underset{v-}{\int }}i\left(V\right)dV,$$ (1)

$${\text{C}}_{\text{SP}}\text{×}\left(\text{F}/\text{g}\right)={\text{C}}_{\text{cell}}/\text{m},$$ (2)

where *C* denotes the measured capacitance for a two electrodes cell and *m* labels the total mass of the active material of electrode. It is clear from the figure that a nanocomposite (1:3) give high specific capacitance. Specific capacitances of all three composites were estimated using Eqs 1, 2 and results are compared in Table 3.

At a higher scan rate, all the charges accumulate at the surface of the electrode material. But at the lower scan rate, ions have ample time for their diffusion in the bulk part of the material to accumulate larger amount of charges and hence results in higher specific capacitance (Rafique et al., 2017a; Rafique et al., 2017b), as shown in Figure 3D.

**FIGURE 4:**
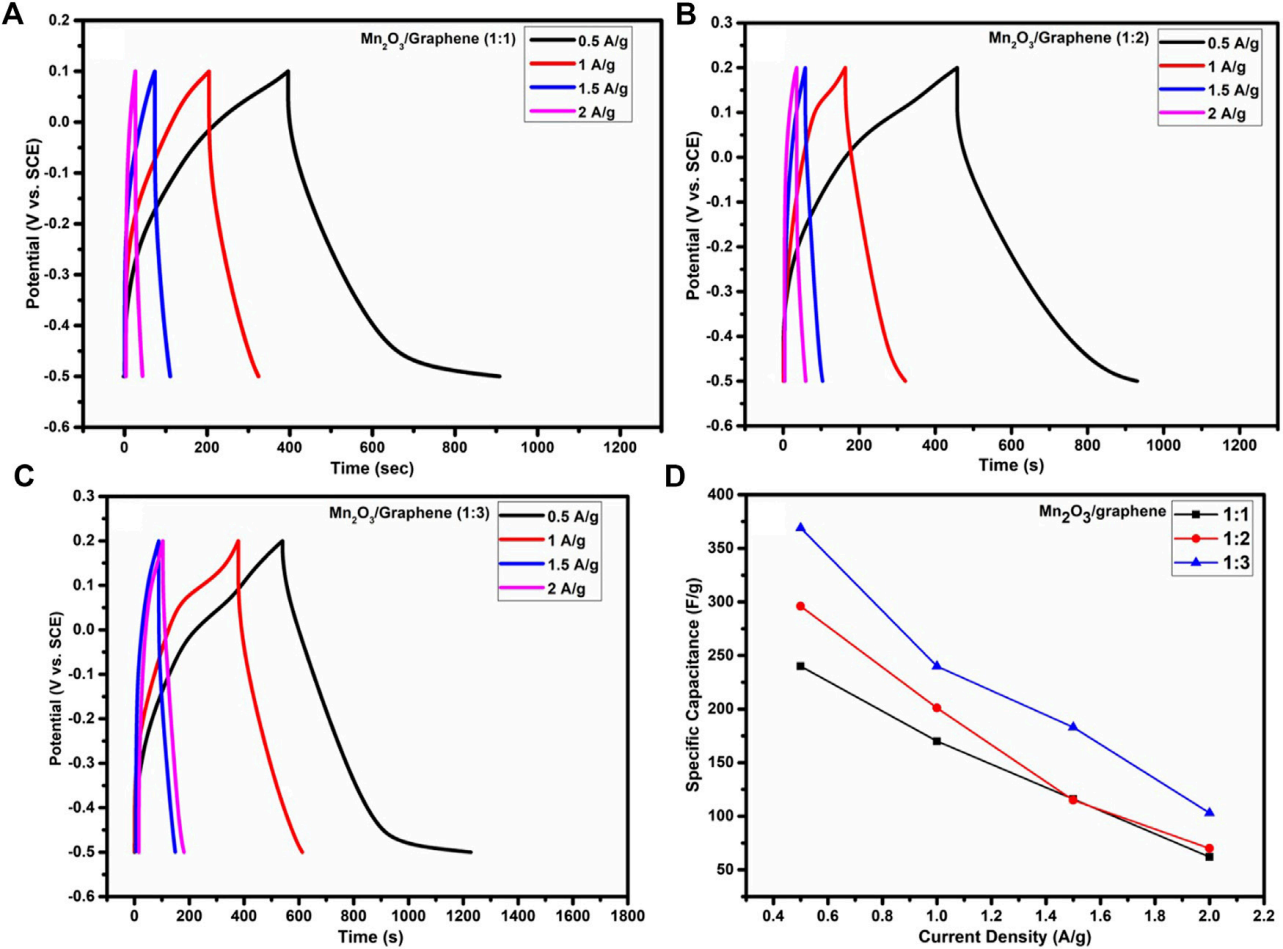
**(A–C)** GCD plots of Mn_2_O_3_/graphene composite samples with 1:1, 1:2, and 1:3, respectively, at 0.5–2 A/g current densities and **(D)** comparison of specific capacitance as a function of current density for all ratios.

The GCD analysis for all three given samples A, B, and C (namely, 1:1, 1:2, and 1:3, respectively) at different current densities in 1 M solution of K_2_SO_4_ electrolyte, as depicted in Figures 4(A–C). The specific capacitances are compared for all three composites, as shown in Figure 4D, and it is clear that as the amount of the graphene increases, current density of the composite material also increases and hence specific capacitance (Rafique et al., 2017b). This also shows that the synergic effect of a carbonaceous material and a metal oxide which gives higher specific capacitance. It is observed that specific capacitance exhibits the decreasing trend with increasing current densities because of an insufficient Faradic reaction, as at a higher scan rate, electrolyte ions have very short time for diffusion in part of the bulk of the electrode material. The comparison of specific capacitance against different current densities is shown in Table 2.

A sinusoidal potential is applied to measure the current that passes through an electrochemical cell and an AC voltage is used to measure the electrochemical impedance which depicted the AC current along with excitation potential including its harmonics (Scully et al., 1993). The pseudo-linear behavior of the cell can be recorded by applying a small value of an excitation signal, but a pseudo-linear response arises at the same frequency with a phase-shift of the applied potential (Láng, 1993).

The Nyquist plot of EIS is a complex plane plot between real and imaginary parts of impedance. The magnitude of complex impedance is represented by a vector length *|Z|*. The data for EIS can be demonstrated by equivalent circuit models (Mansfeld, 1990). A model is typically based on the electrical instruments and their connection which typically controls the shape of the spectrum of the model. The size of a feature containing by a model is controlled by parameters such as resistance of the resistor. From both these factors, the relation between the model and measured EIS spectrum can be confirmed (Mansfeld, 1990).

In order to find the resistances offered by the electrodes, electrochemical impedance spectroscopic measurement was performed by using a three-electrode setup. EIS of the synthesized Mn_2_O_3_/graphene composite electrode has been measured in 1 M K_2_SO_4_ as an electrolyte with a 10-Hz to 1-MHz frequency range.

**FIGURE 5:**
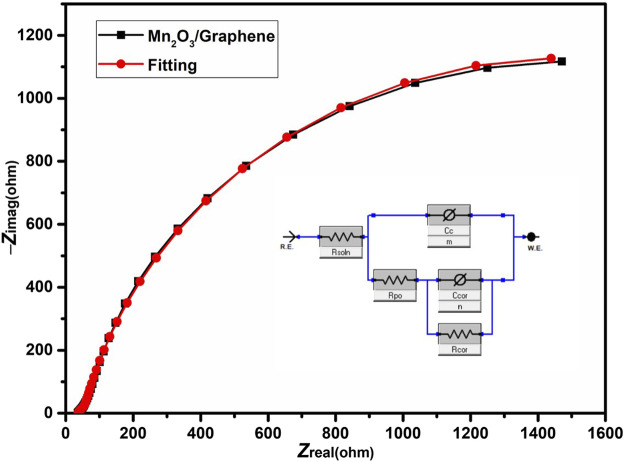
The Nyquist plot of electrochemical impedance spectroscopy of the Mn_2_O_3_/graphene composite material.

The Nyquist plot of this measurement is shown in Figure 5. The Nyquist plot of the Mn_2_O_3_/graphene electrode in the high-frequency region exhibits a semicircle and in the low frequency region a straight line, which reveals the capacitive behavior of the material. The equivalent circuit model used to fit the Nyquist plot is shown as an inset in the Figure 5. The equivalent circuit is composed of solution resistance R_S_, coating capacitance C_C_, a constant-phase element (CPE) exponent *m*, pore resistance R_por_, corrosion “double layer” capacitance C_cor_, a (CPE) exponent *n*, and corrosion resistance R_cor_ of the Mn_2_O_3_/graphene composite electrode material (Walter, 1986). In an electrochemical cell, the solution resistance is often a significant factor between the counter and the reference electrode which is compensated by a modern three electrode setup (Walter, 1986). However, the solution resistance between the working electrode and the reference electrode must be examined during the cell modeling. The resistance of an ionic solution depends on different factors, such as the geometry of the area, ionic concentration, temperature, and type of ions in which current is carried. This resistance for carrying a uniform current, the bounded area A, and the length *l* is defined as follows (Kendig and Scully, 1990):

$$R=\rho \frac{l}{A}.$$ (3)

The solution resistance of the prepared electrode is almost 36 Ω, which shows low equivalent series resistance (ESR) of the synthesized material. The value of R_por_ of the prepared composite material is almost 43.9 Ω shows that ion can easily intercalate and de-intercalate in the pores. In EIS experiments, capacitors often act like a constant-phase element (CPE) and do not behave ideally (Instruments G, 2007). The impedance of a CPE has the following form:

$$Z=\left(1/Yo\right)/{\left(j\varpi \right)}^{\alpha },$$ (4)

where the abovementioned equation explicate a capacitor, the constant Y_0_ = C demonstrates the capacitance and *α* = 1 shows the exponent, and α is less than one for the CPE (Mansfeld, 1990).

**FIGURE 6:**
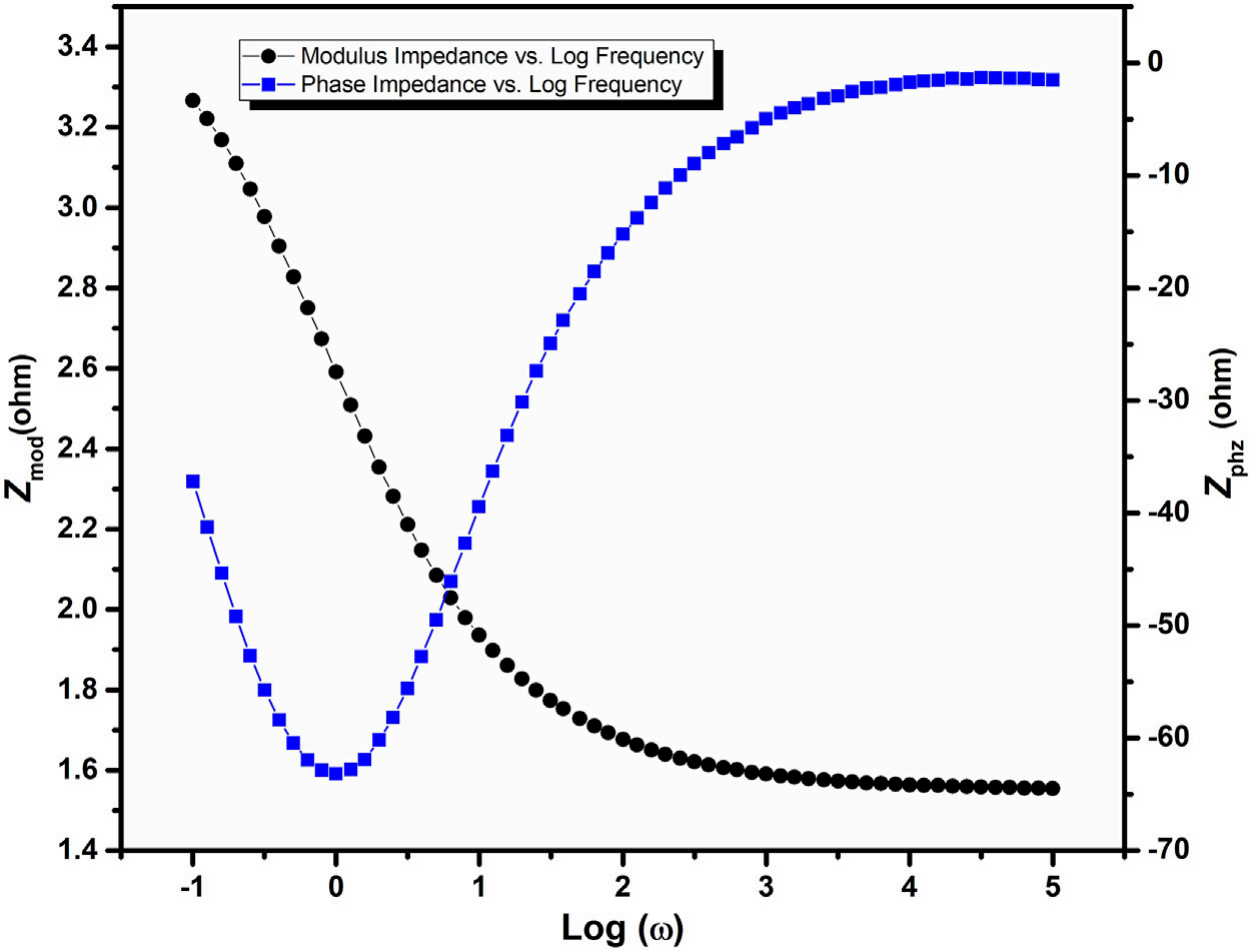
Bode plot of EIS of the Mn_2_O_3_/graphene composite material.

The Bode plot of Mn_2_O_3_/graphene composite is shown in Figure 6. The Bode plot gives the direct readings of the material at low resistance. It is of frequency explicit and is used for the phenomena that can not be investigated through the Nyquist plot. The advantage of this plot is that all information of a single component can be easily understood by the Bode plot. In the Bode plot, shown in Figure 6, the log of frequency is taken along the *x*-axis, while the phase-shift and absolute values of the impedance (|Z| = Z_0_) are taken along the *y*-axis. In the abovementioned figure, the Bode plot of Mn_2_O_3_/graphene composite is sketched using the logarithmic scale. By changing the frequency of the as-prepared composite, the magnitude and the phase angle is obtained. The phase angle tells about the behavior of the supercapacitor.

The pseudocapacitve behavior can be clearly evident from the charge discharge cycles which have increased the specific capacitance, as shown in Table 1. It is also observed that as we increase the content of graphene, current density increases, and hence we have higher specific capacitance. It is also clear from both Table 2 (from GCD) and Table 3 (cyclic voltammetry) that composite three (1:3) gives the higher specific capacitance from other two composite. There might be some reasons behind high capacitance of the composite with 1:3 in comparison to 1:1 and 1:2. For instance, a large surface area is resulted when the ratio of graphene is increased and also the unique structural properties of graphene results into more accumulation of electrolytes which resultantly exhibit high capacitance (Han et al., 2020).

**TABLE 1:** Structural parameters extracted from the X-ray diffraction data and Rietveld profile refinement of Mn_2_O_3_ XRD profile.

Structural results					
Bravais lattice	Space group	Atoms	Wyckoff positions		
			X	Y	Z
Cubic	Ia-3	Mn1	0.25000	0.25000	0.25000
Lattice parameters	Volume unit cell	Mn2	0.97000	0.97000	0.97000
a = b = c = 9.410913 Å	833.48017 Å3	O	0.38500	0.38500	0.38500
Discrepancy factors (%)					
RF		Rexp	RWP	χ2		
4.3164		6.690	8.650	1.67		

**TABLE 2:** Comparison of specific capacitance at different current densities.

Material	Ratio	Electrolyte	Current densities (A/g)	C_s_ (Fg^−1^)
Mn_2_O_3_/Graphene composite	1:1	1molar K_2_SO_4_	0.5, 1, 1.5, 2	178, 119, 75 and 46
	1:2			306, 237, 172 and 70
	1:3			369, 296, 193 and 83

**TABLE 3:** Comparison of cyclic voltammetry (CV) results of Mn_2_O_3_/graphene composites.

Scan rate (mV/s)	Electrolyte	Material (Mn_2_O_3_/graphene) specific capacitance (F/g)		
		Ratio 1:1	Ratio 1:2	Ratio 1:3
5	1M K_2_SO_4_	271	321	391
10	1M K_2_SO_4_	243	251.6	309
20	1M K_2_SO_4_	196	197	261
50	1M K_2_SO_4_	131	138.3	182
100	1M K_2_SO_4_	88	94	93

## Conclusion

An Mn_2_O_3_/graphene composite was synthesized with a simple, low-cost, environmental friendly facile method. Herein, the sol-gel–derived Mn_2_O_3_ resulted in a nanoporous structure of prepared materials. The high porosity of the materials provides a better electrolyte-electrode interface with a larger specific area that was desirable for energy storage applications. Composites of Mn_2_O_3_/graphene were prepared with sonication plus electric motor blending. The method used is simple and cost-effective that can be scaled-up for industrial scale production. Mixing of composites by sonication and mechanical blending resulted in exfoliation of graphene and uniform mixing of composite electrode materials. The XRD pattern confirmed a cubic structure of Mn_2_O_3_ having a crystallite size of about 22.7 nm. The surface morphology of Mn_2_O_3_ investigated by SEM exhibited nanoporous microstructures. SEM micrographs of the composite revealed the uniform mixing of Mn_2_O_3_ with exfoliated graphene sheets. Furthermore, the electrochemical data of different mass ratios of Mn_2_O_3_/graphene composites obtained from GCD and CV showed an improved capacitance in the applied potential window. This report provides an effective and simple route to synthesize Mn_2_O_3_/graphene composites with an enhanced electrochemical performance for supercapacitor applications. Sample with an Mn_2_O_3_/graphene ratio of 1:3 has highest specific capacitance of up to 391 Fg^-1^ at a scan rate of 5 mV/s due to a large surface area and high conductivity provided by increased amount of graphene. This research work provides an effective and simple way to prepare manganese oxide Mn_2_O_3_/graphene composites with high electrochemical performance for supercapacitors. Samples of Mn_2_O_3_/graphene with ratios 1:1, 1:2, and 1:3 have specific capacitance of 271, 321, and 391 Fg^-1^, respectively, at a scan rate of 5 mV/s. The capacitance of composite exhibited an increasing trend with the increasing graphene concentration. These results lead to the conclusion that composite is useful electrode material for super capacitor applications.

## Data Availability

The original contributions presented in the study are included in the article/Supplementary Material; further inquiries can be directed to the corresponding authors.
